# A case report of reversible ischemic MRI changes and discussion of possible link to kratom use

**DOI:** 10.1016/j.ynirp.2025.100261

**Published:** 2025-04-17

**Authors:** Danah Bakir, Christian Konopka, Sindhu Pisati, Syed Shah, Shashi Maryala, Andre Catalano, Faisal Ibrahim, Hesham Allam

**Affiliations:** Southern Illinois University School of Medicine, USA

## 1 Introduction

Kratom (*Mitragnyia speciosa*) is an herbal supplement native to Southeast Asia with opioid properties commonly used to treat chronic pain, anxiety, mood disorders, and opioid withdrawal [113]. Literature has suggested that kratom has unique dual properties; it acts as both a stimulant at lower concentrations, similar to cocaine, and a depressant at higher concentrations, similar to opioids (Castillo et al., 2017; Sethi et al., 2020; Largeau et al., 2020). Two main active components of kratom, mitragynine and 7-hydroxymitragynine, contribute to the analgesic effects (Eggleston et al., 2019). Recent studies have demonstrated that at lower concentrations, these alkaloids show higher affinities for adrenergic receptors, such as α1 and α2. At higher concentrations, they primarily bind to opioid receptors, lead to the depressant and analgesic effects. In addition to their activity at mu-opioid receptors, recent studies have demonstrated that these alkaloids also acts as a competitive antagonists at delta and kappa opioid recepters [4Another alkaloid in kratom, including corynantheidine, has also been found to exhibit strong affinities for adrenergic α1D and α2A receptors, further elucidating potential mechanisms of stimulant effects at lower dosing (Obeng et al., 2020).

Given the complex pharmacological nature of nature of kratom alkaloids involving modulation of both adrenergic and opioid receptor activation, the full extent of kratom's clinical effects has not yet been fully realized. Case reports have been described that associate kratom use with multiple complications such as adverse cardiovascular effects, hepatotoxicity, and coma (Castillo et al., 2017; Singh et al., 2018). Literature is especially limited on how this substance affects the central nervous system. Prior case reports have described kratom use causing seizures as well as posterior reversible leukoencephalopathy syndrome, or posterior reversible leukoencephalopathy syndrome (PRES) (Castillo et al., 2017; Alsarraf et al., 2019). Here, we report a case of excessive kratom use as a potential cause of acute (Diffusion-Weighted Imaging) DWI changes with associated Apparent Diffusion Coefficient (ADC) drop-out representing true cytotoxic edema that was reversible on follow-up MRI.

## 2 Case presentation

We report the case of an individual in their early 20s with a history of anxiety, depression, traumatic brain injury status-post craniotomy, substance use disorder including benzodiazepines, opioids, and kratom use that presented with unresponsiveness, shallow breathing, and possible seizure-like activity. The patient was found on the living room floor and was noted to be “pale and clammy”. The patient does not have a known history of neurological disorders, including epilepsy. Of note, there was drug paraphernalia equipment at the scene where the patient was found to be altered including burnt foil, thus the suspicion for substance ingestion is high, especially opiates according to bystanders at the scene. Despite the administration of at least three doses of Narcan (naloxone), the patient showed only minimal improvement with no reversal of symptoms. Systolic blood pressure was found to be mildly elevated in the 140–149 mmHg, pulse at 115 beats per min (bpm), and respiratory rate was typical at about 20 breaths per minute. EKG at admission showed sinus tachycardia. Blood glucose was 3.6 mmol/L at the scene. Neurological findings at presentation included bilateral constricted pupils, right gaze deviation, and irregular spastic-like movements on the left with clenching of the left fist. The patient was eventually intubated for airway protection upon arrival at the emergency department. Notably, the patient's mother provided a detailed account of the patient's extensive kratom use on a daily basis for many years to help with his anxiety, as well as increased recent kratom use prior to presentation due to a life stressor. It is unclear exactly how much kratom the patient was ingesting.

CT head at arrival revealed stable encephalomalacia of the left frontal lobe from left craniotomy and no evidence of acute territorial infarction, hemorrhage, or mass effect. Upon presentation to the emergency department, there were multiple disparities regarding the possible seizure-like activity on the left side. The patient was found to be hypotensive and hypoglycemic with concerns for an underlying infection considering he had leukocytosis (23.9 × 10^9^/L), lactic acidosis (4.7 mmol/L), and elevated procalcitonin (9.97 ng/mL). Urine toxicology screening was positive for benzodiazepines, likely from the lorazepam dosages given at the emergency department for concerns for seizure activity, and cannabinoids, but nothing else in the panel was detected, as outlined in Table 1. Lumbar puncture revealed normal WBC and RBCs **(<** 1 cell/μL), normal protein (30 mg/dL), normal IgG index (2.3), and slightly elevated glucose (4.5 mmol/L mg/dL). Serum glucose is normal (6.2 mmol/L mg/dL). CSF: serum glucose ratio is normal (0.7). CSF cultures including HSV, VDRL, and Lyme were negative. CSF ACE and myelin basic protein were negative.(See Fig. 1a,1b)

**Table 1:** Summary of Urine Toxicology Results. Benzodiazepines and cannabinoids returned unconfirmed positives (Unconf +). Asterisks (∗) indicate results near the assay's detection limits. Most substances showed no drug levels (NDL).

Test	Result
Blood Ethanol	NDL
Tricyclic Antidepressant scr serum	NDL∗
Amphetamine screen	NDL
Barbiturate screen	NDL
Benzodiazepine screen	Unconf +
Cannabinoids screen	Unconf +
Cocaine screen	NDL
Propoxyphene screen	NDL
Methadone screen	NDL
Methaqualone screen	NDL
Opiates screen	NDL∗
Phencyclidine screen	NDL∗
Urine ethanol screen	NDL

Given the history of brain injury, lab derangements, history of substance abuse, and high possibility for sepsis, any seizure activity was likely to be provoked. Levetiracetam **(**Keppra) was initiated at a loading dose of 1000 mg administered intravenously over 100 mL at 400 mL/h as a one-time dose and an EEG was obtained, shown in Fig. 1. The patient was started on broad-spectrum antibiotics to cover the possibility of meningitis, as well as a maintenance dose of Keppra to prevent seizures.

A couple of days into admission, the patient was noted to have Babinski sign on the right lower extremity as well as significant right upper extremity weakness when examined off sedation that were not present during admission, concerning for acute stroke. A stroke workup was subsequently performed including MRI and CT angiogram, as well as an echocardiogram and EKG. MRI brain, shown in Fig. 2, revealed small moderate areas of abnormal diffusion-weighted imaging changes with associated ADC drop out in the posterior frontal lobes bilaterally, representative of true diffusion restriction consistent with cytotoxic edema due to infarction. There is also stable encephalomalacia bilaterally of the superior frontal gyri, larger on the left compared to the right, a consequence of the patient's history of traumatic head injury with subsequent craniotomy.

**Fig. 1a:**
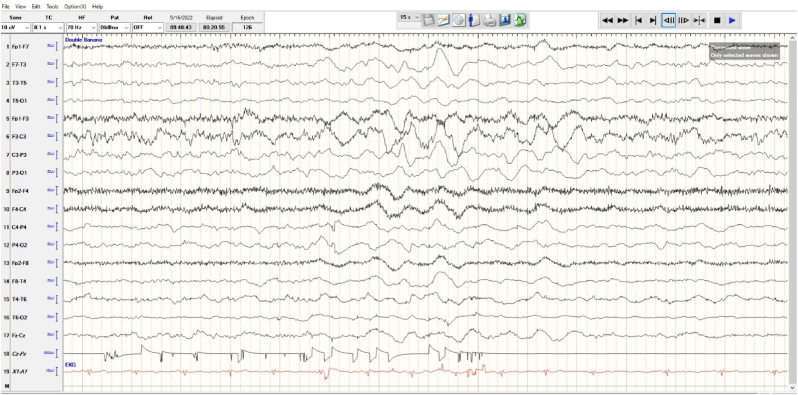
EEG showing generalized slowing maximum in the left frontal region consistent with diffuse severe encephalopathy and cortical dysfunction in the left frontal region. Asymmetry noted in the left frontal region as well with increased background consistent with an overlying skull defect in the left frontal region.

**Fig. 1b:**
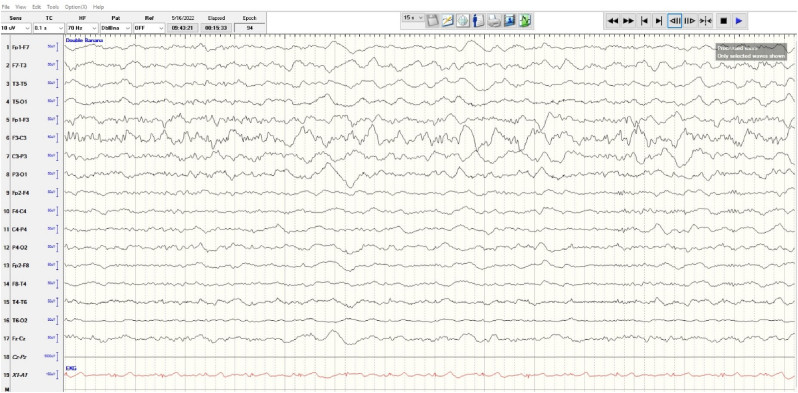
Intermittent rhythmic slow in delta range, maximum in bifrontal region. There were no epileptiform discharges or seizures recorded.

**Fig. 2a:**
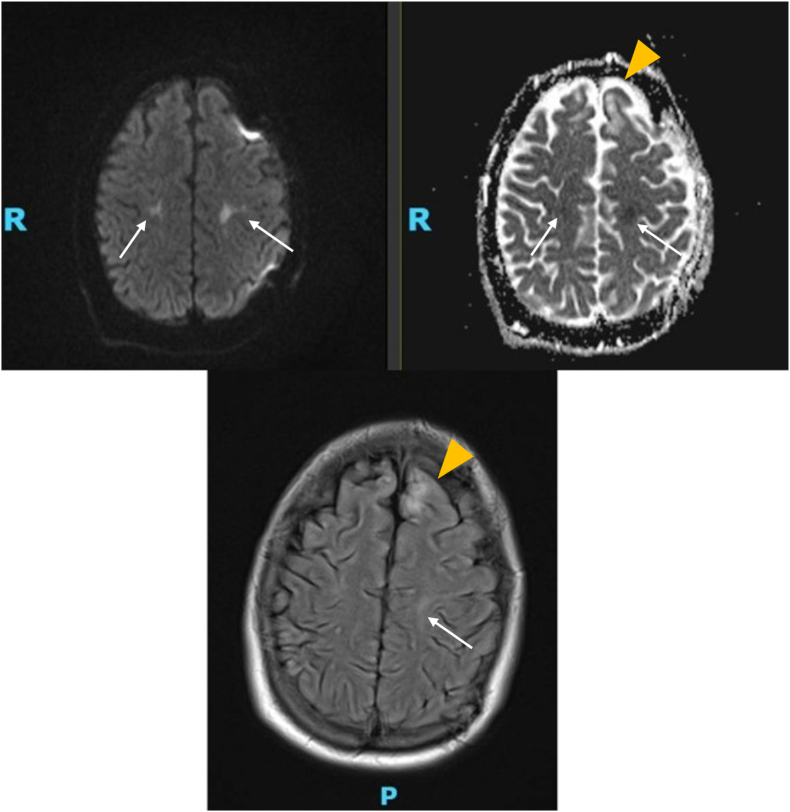
**Left**: DWI sequence. **Right**: ADC sequence. **Bottom**: T2-weighted FLAIR image. Encephalomalacia of the bilateral superior frontal gyri, larger on the left hemisphere compared to right, due to known history of traumatic brain injury. Moderate areas of subcortical or juxta-cortical white matter abnormalities high in the posterior frontal lobes bilaterally on DWI with associated ADC dropout representative of true diffusion restriction consistent with acute ischemic infarct**.** Images were obtained using a 1.5T MRI scanner. DWI acquisitions were trace-based with 3-mm slice thickness and 0.4-mm interslice gap. ADC sequences were acquired with identical parameters. T2-FLAIR images were acquired with a 5-mm slice thickness and 0.65-mm interslice gap.

**Fig. 2b:**
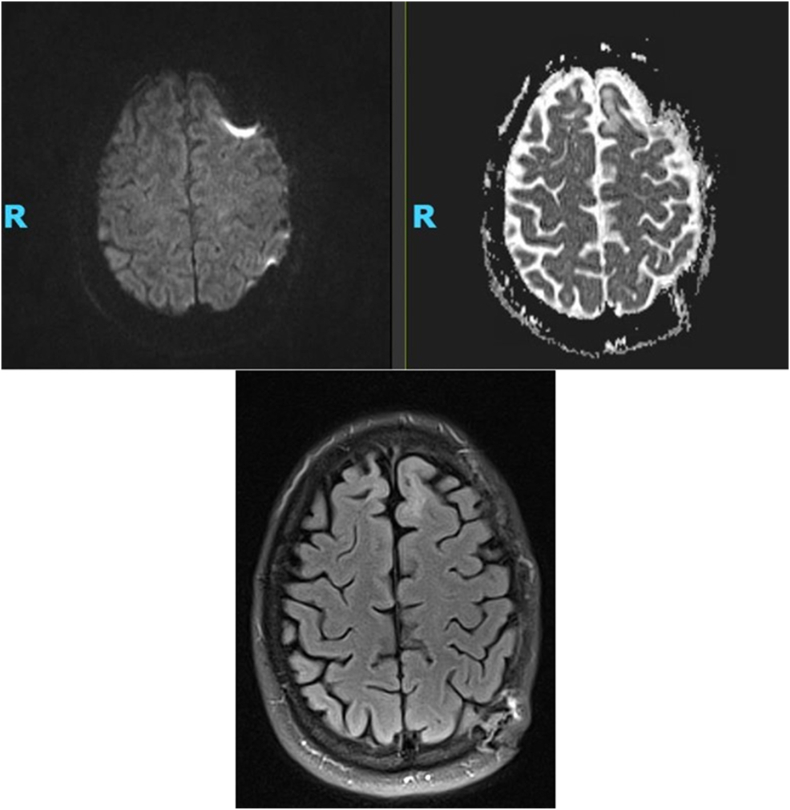
**Left**: DWI sequence. **Right**: ADC sequence. **Bottom**: T2-weighted FLAIR image. Resolution of hyperintensities on DWI with no new diffusion restriction, indicating no acute intracranial ischemic infarct. No pseudonormalization of the ADC sequence and no residual FLAIR hyperintensities, consistent with complete reversal of ischemic MRI changes. Follow-up images were acquired on a 3T MRI scanner. DWI acquisitions were trace-based with 3-mm slice thickness and 0.4-mm interslice gap. ADC scans were performed with identical parameters. T2-FLAIR images were acquired with a 3-mm slice thickness and 0.4-mm interslice gap. Quantification of DWI signal changes and ADC values is not feasible due to differences in MRI acquisition parameters and scanner calibration across studies, which precludes longitudinal quantification.

Intracranial CT angiogram was unremarkable and revealed no concerns for intracranial occlusion, stenosis, or aneurysm. Carotid CT angiogram revealed no occlusion, stenosis, or dissection in the major neck arteries. A transthoracic echocardiogram revealed an ejection fraction of 62 %, mild concentric left ventricular hypertrophy, mildly increased ventricular wall thickness, and mildly dilated right atrium. The rest of the stroke workup was unremarkable.(See Fig. 2a,2b)

He was loaded with a full dose of aspirin 325 mg alongside permissive hypertension in the first 24–48 h with a systolic blood pressure goal of <220/110 mmHg, then continued on a smaller aspirin dose of 81 mg daily. A follow-up repeat MRI conducted about two months later revealed the resolution of prior anomalies, thus indicating the reversal of initial ischemic changes.

## 3 Discussion

Kratom's therapeutic analgesic and opioid-like effects have been implicated as an inexpensive alternative to evidence-based treatment for opioid withdrawal (Valenti et al., 2021; Singh et al., 2018). However, its rising popularity is met with safety concerns. Kratom is being recognized as a potential substance of abuse given its stimulant and depressive activity. One of its primary components, mitragynine, exhibits a multitude of effects including sympathetic and serotonergic activity, which can potentially lead to vasoconstriction or vasculitis (Valenti et al., 2021; Burke et al., 2021; Kerrigan and Basiliere, 2022). Not enough information about the long-term effects of kratom ingestion, especially on CNS health, is known to make appropriate recommendations on the consequences of this drug. It is important to consider that kratom may also demonstrate adverse synergistic or additive effects with other drugs as well, such as amphetamines (Castillo et al., 2017; Kerrigan and Basiliere, 2022). Due to the limited regulation of this drug, there is a significant risk of contamination (Kerrigan and Basiliere, 2022). This risk is particularly relevant in patients with a history of polysubstance abuse.

Use of this drug has recently gained popularity in the West, as many seek a “natural” alternative to opioids is desirable for treatment of opioid withdrawal symptoms, chronic pain, and mood disorders (Burke et al., 2021). However, the US Drug Enforcement Administration (DEA) has labeled kratom as a “drug of concern” due to its steady increase in fatalities from drug overdose (Burke et al., 2021; Suhaimi et al., 2016; Michael, 2019). This has drawn attention to kratom as a significant public health issue and safety concern, especially considering its lack of regulation and ease of accessibility. As recreational and medicinal kratom use in the United States becomes more prevalent, and given the lack of regulation concerning its acquisition, it is important for neurologists to acknowledge the history of kratom use, the pathological mechanisms underlying its neurotoxic effects on the brain and intracranial blood vessels, and any toxic neurological effects that may result from its regular consumption.

Although kratom use was not directly confirmed through toxicological testing in this case, its involvement was supported by the patient's extensive history of chronic use as reported by the mother. Symptoms associated with kratom toxicity have been reported to include seizures, respiratory depression of varying severity, QTc prolongation and other cardiac abnormalities, delirium with altered levels of consciousness, and muscle rigidity accompanied by spastic-like movements [12]. These manifestations are consistent with this patient's presentation of seizure-like activity, shallow breathing, and altered mental status. Animal studies have shown that naloxone only partially reverses the analgesic effects of kratom, reflecting the unique interaction of its alkaloids with the mu-opioid receptor. This aligns with the patient's clinical presentation, as the administration of at least three doses of naloxone resulted in only minimal improvement with no reversal of symptoms [12]. Other case reports have described how kratom use can lead to seizures and PRES (Castillo et al., 2017; Alsarraf et al., 2019; Suhaimi et al., 2016; Michael, 2019). A presumed mechanism underlying kratom effects is that it can trigger sympathetic effects leading to vasoconstriction and rapid onset severe hypertension that leads to a presentation of PRES, leading to loss of autoregulation and development of vasogenic edema. It is postulated that kratom's effects on the mu-subtype opioid receptors could also be the mechanism underlying PRES (Castillo et al., 2017). The specific dose and duration of usage were not reported in these cases. As a result, it is unclear if there is a correlation between the amount of kratom ingestion and subsequent adverse events (Alsarraf et al., 2019). While kratom has been reported as causing seizures and PRES, it is unclear the association between kratom and ischemic brain damage.

A limited number of studies have commented on kratom use and its effects on brain morphology and specifically how it affects the size of cortical and subcortical structures (Singh et al., 2018). Studies have also mentioned that mitragynine may cause cognitive deficits and impaired learning and memory in mice (Kerrigan and Basiliere, 2022). One case report has also described reversible T1 hyperintensities of the basal ganglia in patients with regular kratom use (Valenti et al., 2021). However, to our knowledge, few to no case reports have demonstrated how kratom may cause reversible cytotoxic edema on MRI resembling those of an ischemic stroke. This particular case report is intended to provide insight into the neurotoxic effects of kratom use, possibly due to a vasospastic mechanism. Notably, its association with reversible ischemic MRI changes mimicking acute ischemic stroke.

To our knowledge, this is the first reported case of kratom causing cytotoxic edema, rather than the previously reported cases of vasogenic edema in PRES caused by kratom. We also believe that kratom could also be causing a systemic process due to this patient's MRI demonstrating bilateral insults.

The patient described in this case report showed hallmark signs of an ischemic infarct, as indicated by hyperintense changes in the DWI sequence with corresponding ADC dropout at T2-FLAIR hyperintensity. The abnormal DWI changes in the bilateral superior frontal gyri support a true acute ischemic infarction. Upon follow-up imaging a couple of months following the stroke, the T2-weighted FLAIR image does not reveal any lesions, thus indicating full reversal of ischemic insult. Our report of this patient suggests that kratom use may lead to reversible ischemic MRI changes that resemble an acute ischemic stroke, possibly due to a vasospastic mechanism.

It is crucial to understand the risks associated with kratom use when gathering a relevant history from patients. This is especially true when identifying the etiology of acute ischemic infarctions in younger patient populations, who are more likely to engage in substance use behavior. Additionally, understanding that kratom use can lead to reversible infarctions is important for long-term prognostication of these patients.

## CRediT authorship contribution statement

**Danah Bakir:** Writing – review & editing, Writing – original draft. **Christian Konopka:** Writing – review & editing. **Sindhu Pisati:** Writing – review & editing, Conceptualization. **Syed Shah:** Writing – review & editing. **Shashi Maryala:** Writing – review & editing, Conceptualization. **Andre Catalano:** Writing – review & editing, Supervision. **Faisal Ibrahim:** Writing – review & editing, Formal analysis. **Hesham Allam:** Writing – review & editing, Supervision, Formal analysis, Conceptualization.

## Ethics statement

This case report was prepared in compliance with ethical and publishing standards. As this report describes a retrospective case with no experimental interventions, with less than three patients, it does not meet the DHHS definition of research (45 CFR 46.102(l)) or the FDA definition of clinical investigation (21 CFR 46.102(c)) and therefore was not required to be submitted to SIU Medicine IRB for review and determination. Written informed consent could not be obtained from the patient due to loss to follow-up, despite reasonable efforts to make contact. In accordance with Elsevier's publishing guidelines, all potentially identifying details have been removed to protect patient confidentiality. The included imaging is entirely anonymized and does not contain any identifying marks.

## Funding sources

This research did not receive any specific grant from funding agencies in the public, commercial, or not-for-profit sectors.

## Declaration of competing interest

The authors declare that they have no known competing financial interests or personal relationships that could have appeared to influence the work reported in this paper.

## Data Availability

No data was used for the research described in the article.
